# Supplementary material for: Transcriptional signatures of schizophrenia in hiPSC-derived NPCs and neurons are concordant with post-mortem adult brains
Source: Nat Commun. 2017 Dec 20;8:2225. doi: 10.1038/s41467-017-02330-5 (PMC5738408; doi:10.1038/s41467-017-02330-5)
Supplement: Supplementary file 1 — Supplementary Information [file 41467_2017_2330_MOESM1_ESM.pdf]

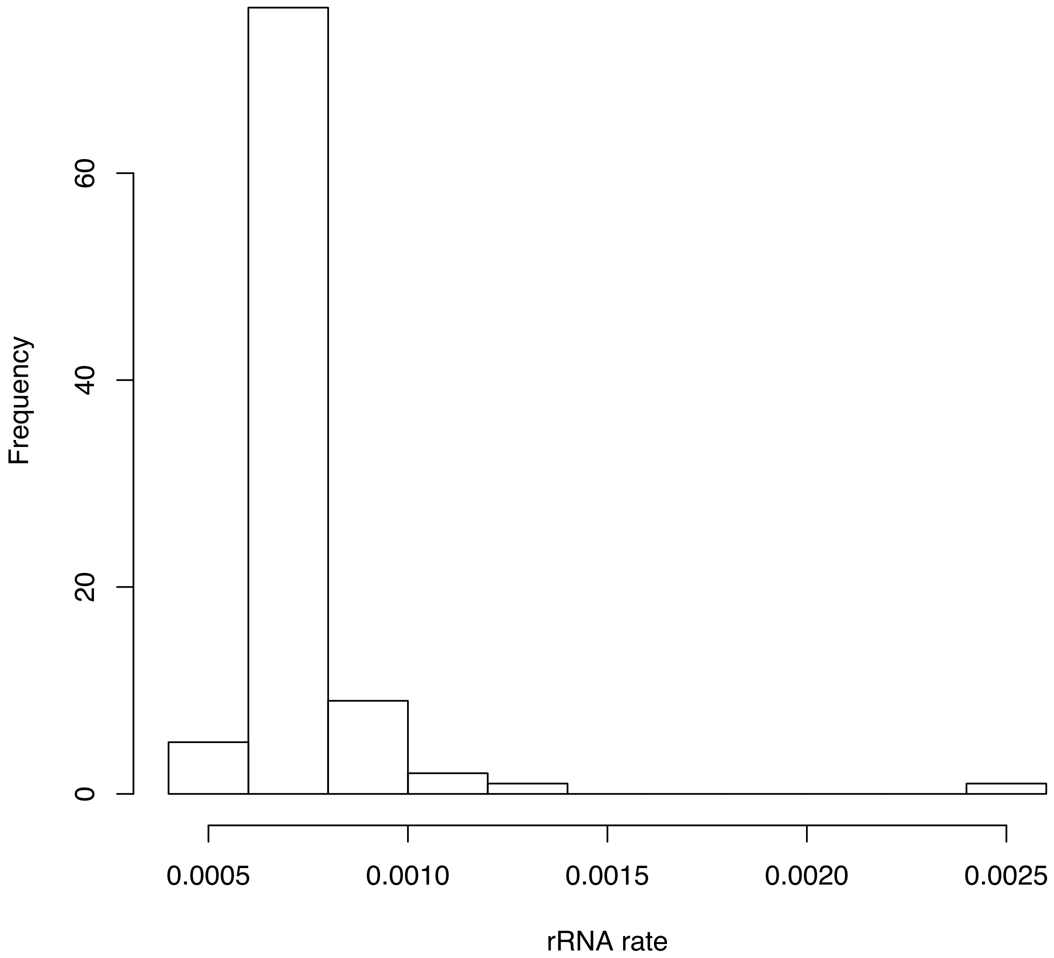

Supplementary Figure 1: Ribosomal RNA rate computed from each RNA-Seq experiment

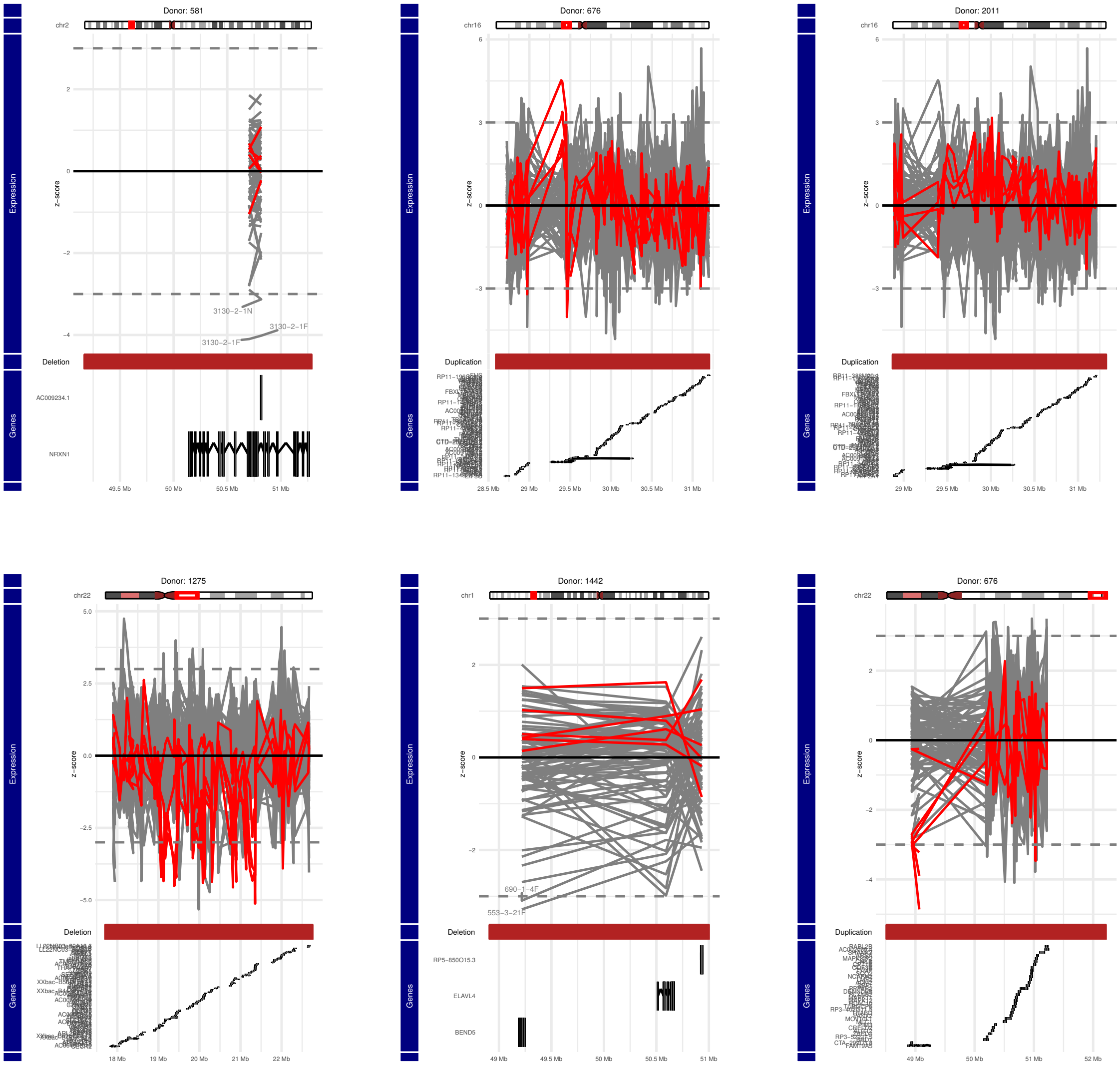

Supplementary Figure 2: Effect of copy number variation on expression of proximal genes. Expression of genes near CNV breakpoints were plotted and z-score of expression of each gene was used to identify expression outliers. Each line presents the expression of the set of genes for individuals with the CNV (red) and without the CNV (grey). Z-scores are plotted at the midpoint of the body of each gene.

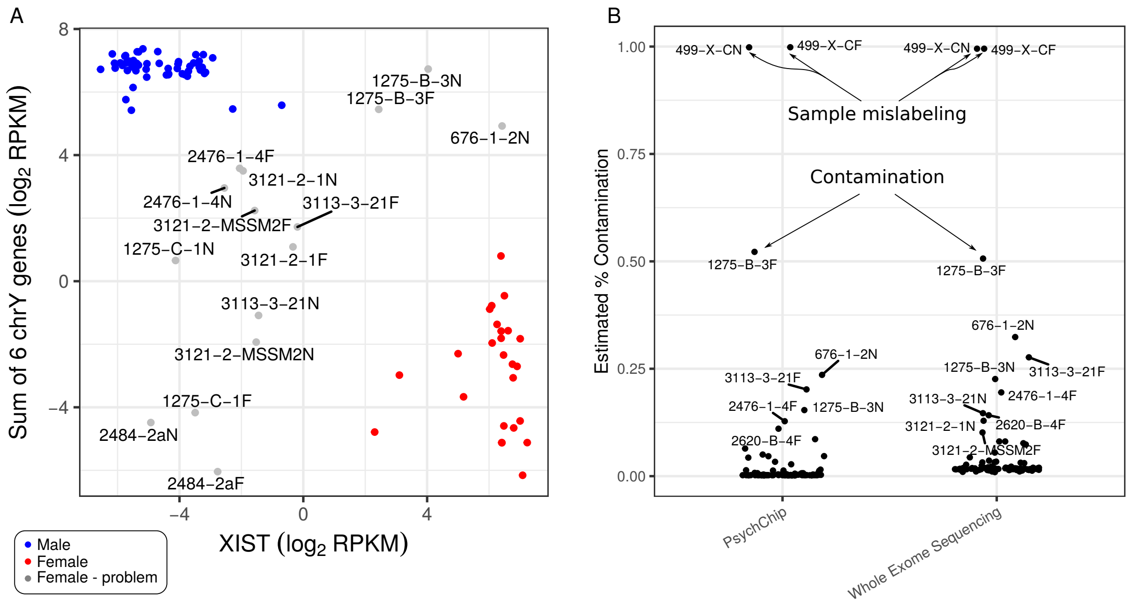

Supplementary Figure 3: Quality control for sex, contamination and mislabeling. A) Check that labeled sex is concordance with gene expression on chrX, and chrY. Plot of the sum of expression of 6 chrY genes (*USP9Y*, *UTY*, *NLGN4Y*, *ZFY*, *RPS4Y1*, *TXLNG2P*) versus expression on *XIST* from chrX. Males (blue) have distinct expression patterns of high chrY and low chrX expression. High quality female samples (red) have high chrX expression and low chrY expression. Problematic samples (grey) have intermediate expression patterns due to problems in X-inactivation, sample mislabeling or contamination involving a male and female sample. These samples were excluded from further analysis. These individuals are not known to have Klinefelter's or other sex chromosome abnormality that would produce this observation. **B**) Contamination analysis using VerifyBamID [1] comparing variants called for each sample from RNA-Seq to variants from PsychChip and whole exome sequencing of the donors. Individual 499 shows a contamination percentage of 100%, recapitulating a known issue with sample mislabeling. Sample 1275−B−3F has a contamination percentage of 50%, consistent with (A) where this sample shows and expression patter intermediate between male and female. This sample is likely contains both male and female RNA.

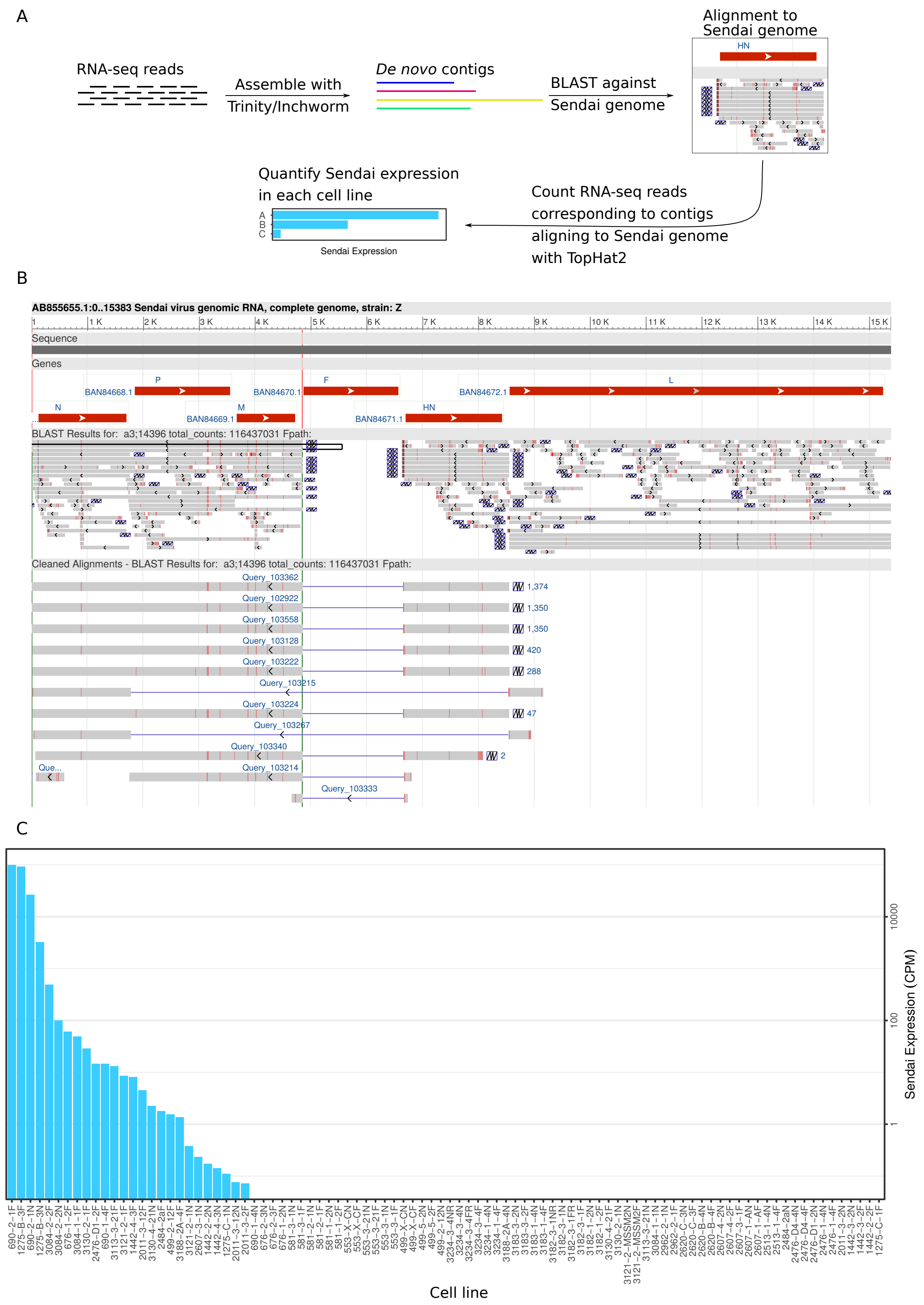

Supplementary Figure 4: Quantifying residual Sendai virus from RNA-Seq reads. **A**) Analysis workflow illustrating *de novo* assembly with Trinity/Inchworm,

aligning contigs to Sendai genome and quantifying Sendai expression for each RNA-Seq experiment. **B**) Plot from NCBI showing results of BLAST alignment to the Sendai virus genome of all *de novo* contigs compiled across all 94 RNA-Seq experiments. Notice that Sendai gene F is not observed in the dataset likely due to the fact that the virus used in the experimental procedure was engineered. **C**) Quantification of Sendai expression in counts per million for each RNA-Seq experiment.

A

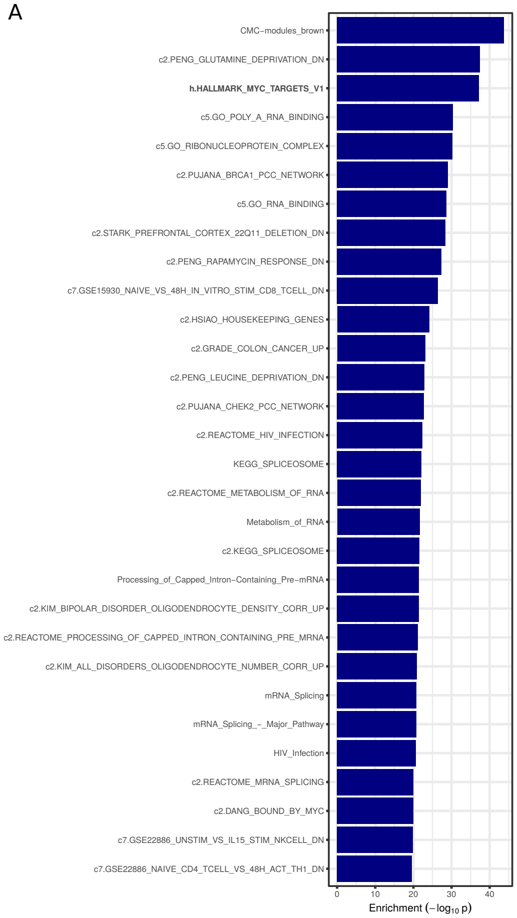

B

	logFC	AveExpr	t	P.Value	qvalue	gene
ENSG00000136997	0.30217081	3.1929949	2.1221004	0.03714721	0.08496842	*MYC*
ENSG00000181449	−0.05616205	8.7614381	−1.0330145	0.30493241	0.28158263	*SOX2*
ENSG00000136826	−0.13880021	0.0752657	−0.8533798	0.39617890	0.32496601	*KLF4*

Supplementary Figure 5: Genes differentially expressed based on residual Sendai virus expression. A) Gene set enrichment based on hypergeometric test for genes with FDR < 5%. **B**) Differential expression results for 3 Yamanaka factors genes used in a Sendai virus vector in the hiPSC reprogramming. *POU5F1* (i.e. *OCT4*) is not expressed at sufficient levels to be included in this analysis.

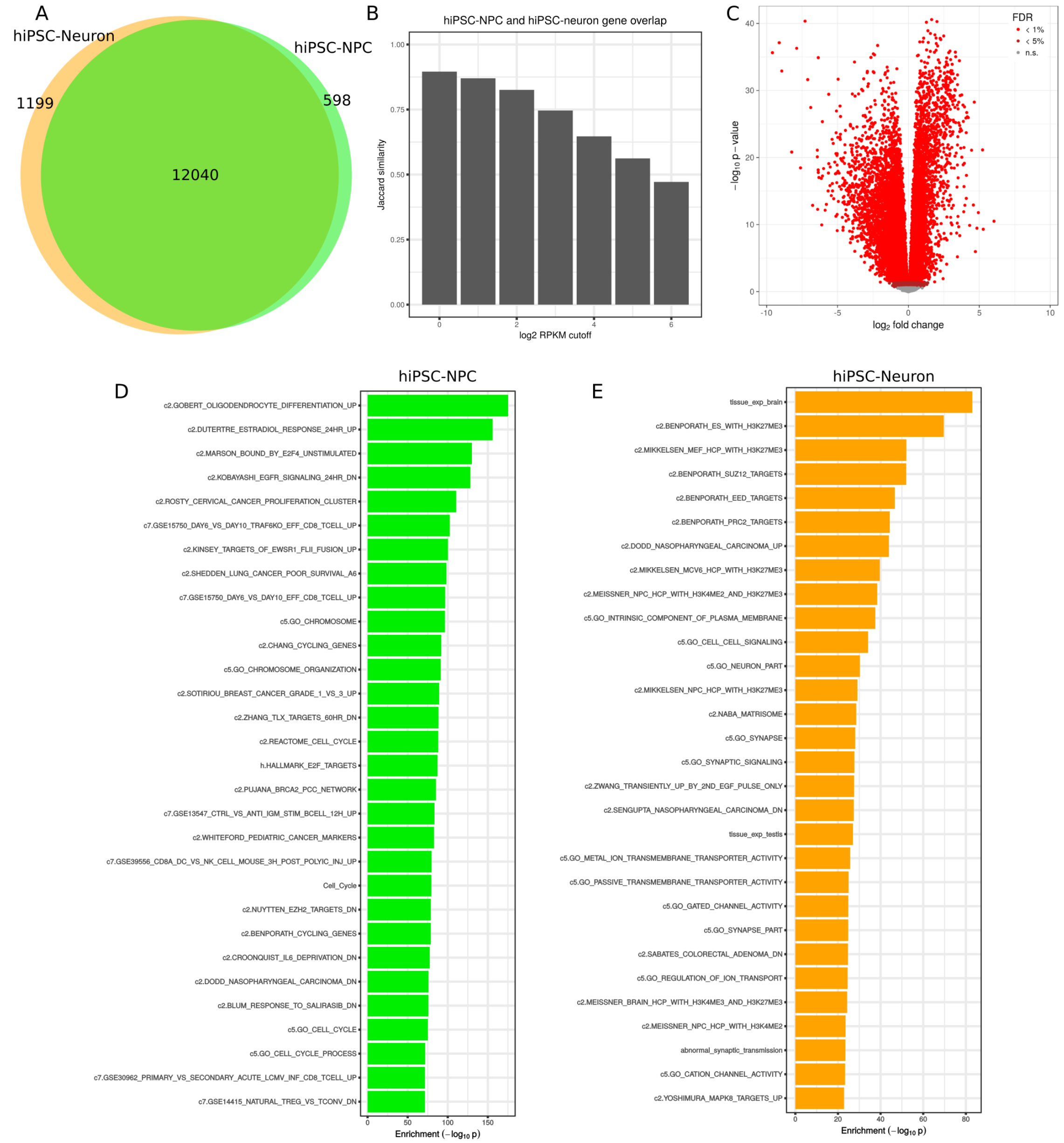

Supplementary Figure 6: Comparing expression patterns in hiPSC-NPC and hiPSC-neurons. **A**) Venn diagram indicating high overlap of genes expressed at $\log_2$ RPKM of 1 in each cell type. **B**) Jaccard similarity between sets of genes that are expressed in each cell type at a level exceeding the expression cutoff on the x-axis. This indicates high overlap between sets of expressed genes. **C**) Volcano plot showing $-\log_{10}$ p-value and $\log_2$ fold change between hiPSC-NPC and hiPSC-neurons. Genes with FDR < 1% are indicated in light red and genes with FDR < 5% are indicated in dark red. Remaining genes are show in grey. **D,E**) Gene set enrichment tests based on hypergeometric test for gene sets in MSigDB for genes with FDR < 1% in D) hiPSC-NPCs and E) hiPSC-neurons.

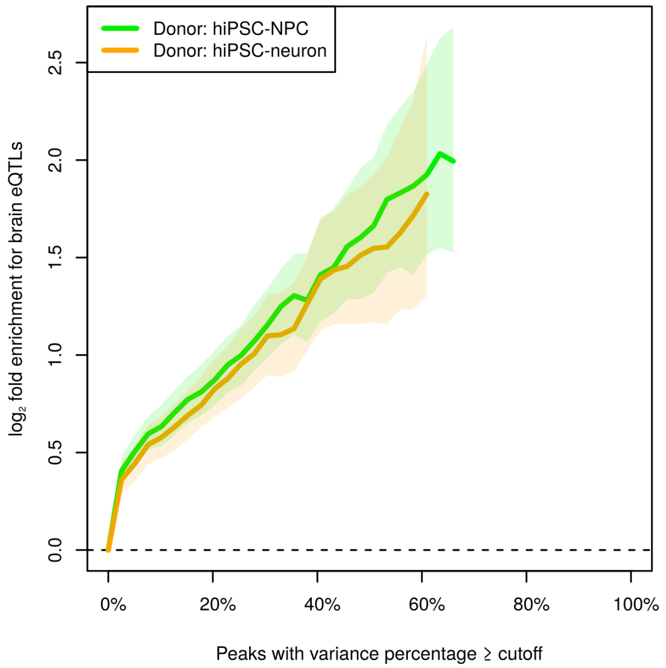

Supplementary Figure 7: Genes with high inter-donor expression variation in hiPSC-NPCs and -neurons are enriched for brain cis-eQTLs. Fold enrichment (log_2) for the 2000 top cis-eQTLs discovered in post mortem dorsolateral prefrontal cortex data generated by the CommonMind Consortium[2] shown for the inter-donor variance component in hiPSC-NPCs and –neurons. Each line indicates the fold enrichment for genes with the fraction of variance explained exceeding the cutoff indicated on the x-axis. Shaded regions indicate the 90% confidence interval based on 10,000 permutations of the variance fractions. Enrichments are shown on the x-axis until less that 100 genes pass the cutoff.

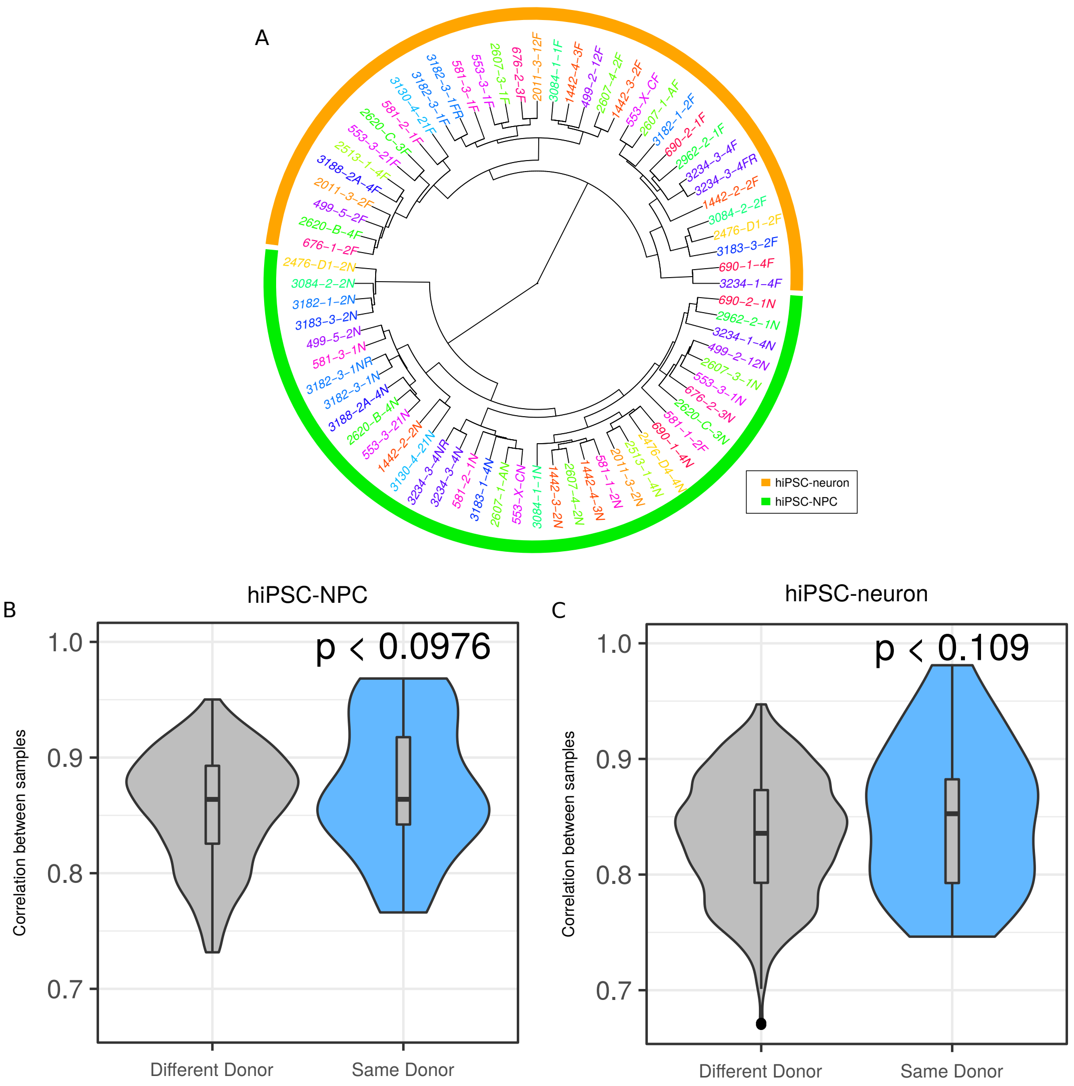

Supplementary Figure 8: Similarity between RNA-Seq samples from the same donor within each cell type. **A**) Hierarchical clustering of RNA-Seq samples before correcting for the two fibroblast cell type composition scores. **B,C**) Correlation between samples from different donors compared to the correlation between samples from the sample donor. P-value indicates one-sided Wilcoxon test. B) Correlations for hiPSC-NPCs before correction. C) Correlations for hiPSC-neurons before correction.

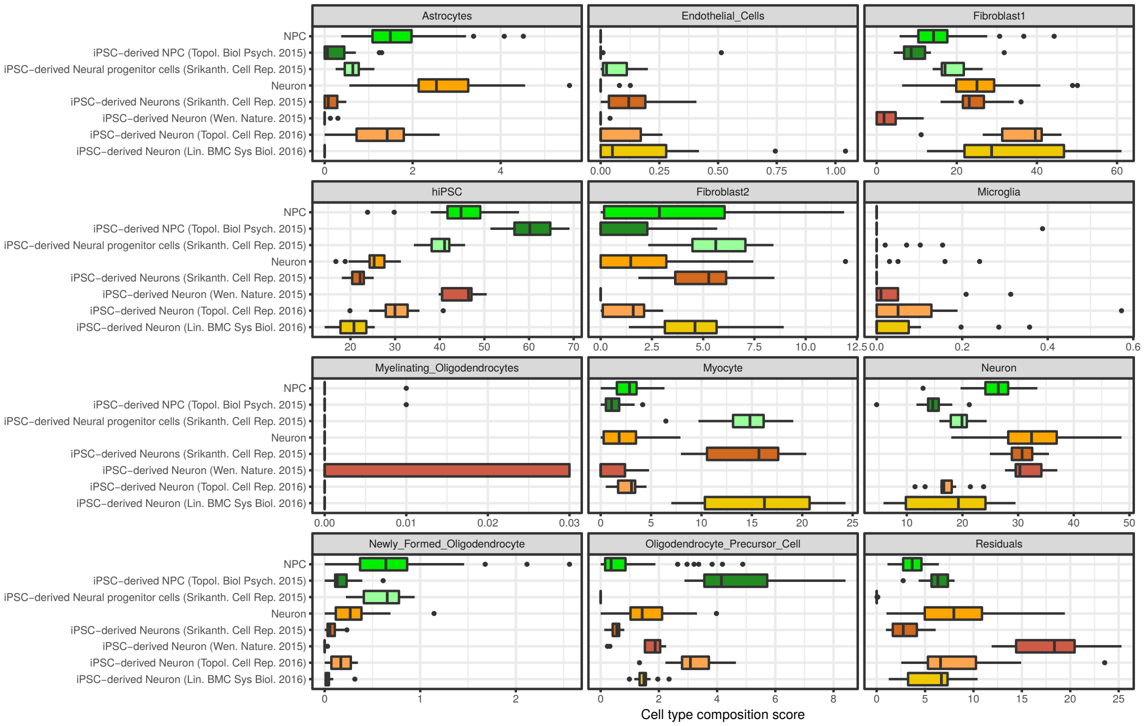

Supplementary Figure 9: Cell type composition scores for current study and hiPSC-NPC and hiPSC-neuron samples from external datasets.

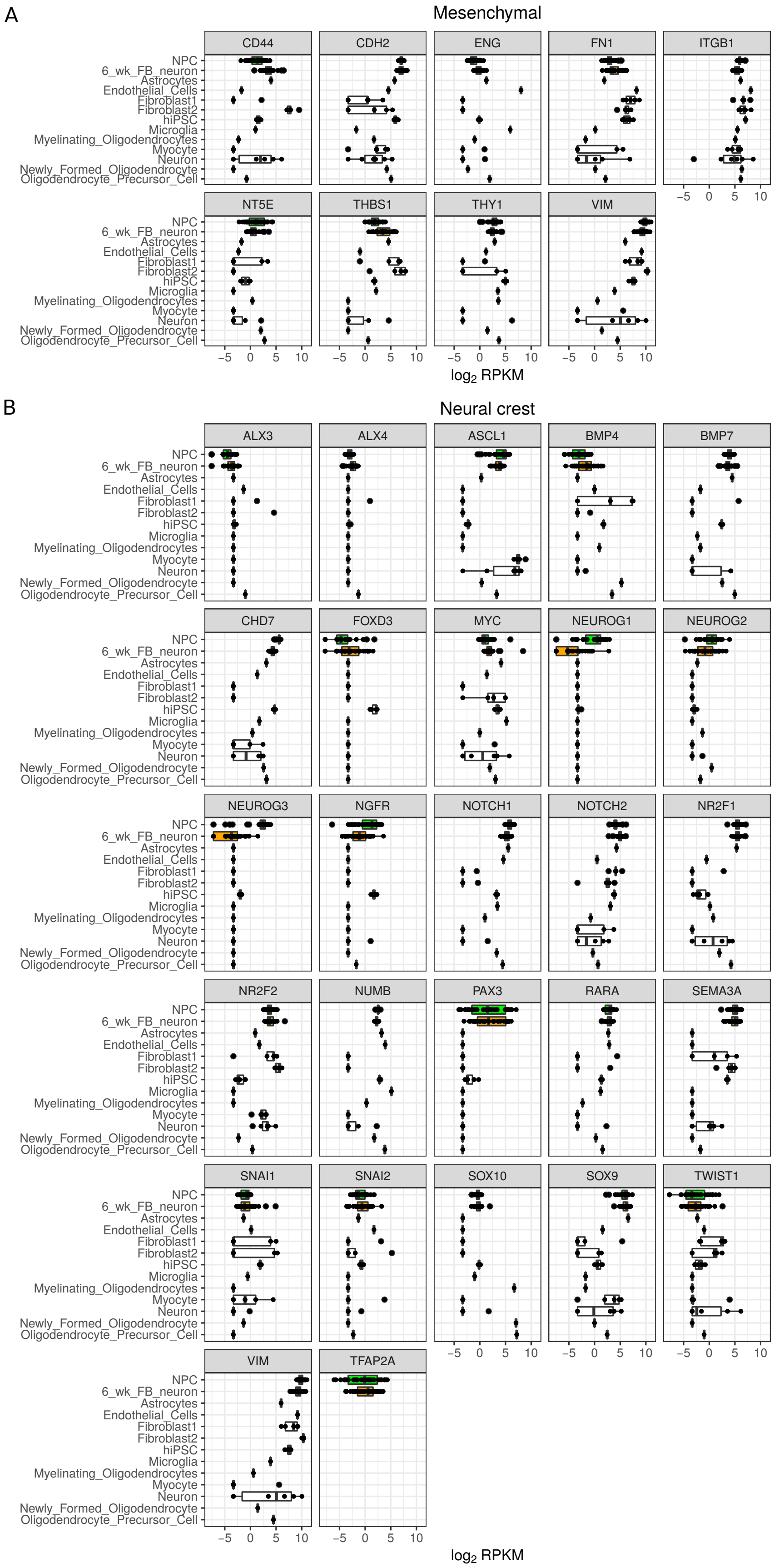
A
Mesenchymal
CD44
CDH2
ENG
FN1
ITGB1
NT5E
THBS1
THY1
VIM
NPC
6_wk_FB_neuron
Astrocytes
Endothelial_Cells
Fibroblast1
Fibroblast2
hiPSC
Microglia
Myelinating_Oligodendrocytes
Myocyte
Neuron
Newly_Formed_Oligodendrocyte
Oligodendrocyte_Precursor_Cell
−5 0 5 10
log2 RPKM
B
Neural crest
ALX3
ALX4
ASCL1
BMP4
BMP7
CHD7
FOXD3
MYC
NEUROG1
NEUROG2
NEUROG3
NGFR
NOTCH1
NOTCH2
NR2F1
NR2F2
NUMB
PAX3
RARA
SEMA3A
SNAI1
SNAI2
SOX10
SOX9
TWIST1
VIM
TFAP2A
log2 RPKM

Supplementary Figure 10. Expression of mesenchymal (top) and neural crest (bottom) markers in hiPSC-NPCs, hiPSC-neurons and across our cell type composition (CTC) signatures.
Plots of selected mesenchymal (*NT5E* (CD73), *VIM, THBS1, CDH2, VTN, FN1, ENG, ITGB1, CD44, THY1*) [3]; http://www.abcam.com/human-mesenchymal-stromal-cell-marker-panel-cd44-cd45-cd90-cd29-and-cd105-ab93758.html) and neural crest (*NGFR* (CD271), *TFAP2A, NR2F1, NR2F2, TWIST1, SNAI1, SNAI2, RARA, ALX3, ALX4, PAX3*, *SOX9, SOX10, MYC, SEMA3A, NOTCH1, NOTCH2, ASCL1, CHD7, FOXD3, NGN1, NGN2, NGN3, NUMB, VIM, BMP4, BMP7*) markers [4,5]; https://www.rndsystems.com/research-area/neural-crest-cell-markers) markers in our hiPSC-NPC and hiPSC-neuron RNA-seq data as well as the CTC reference signatures.

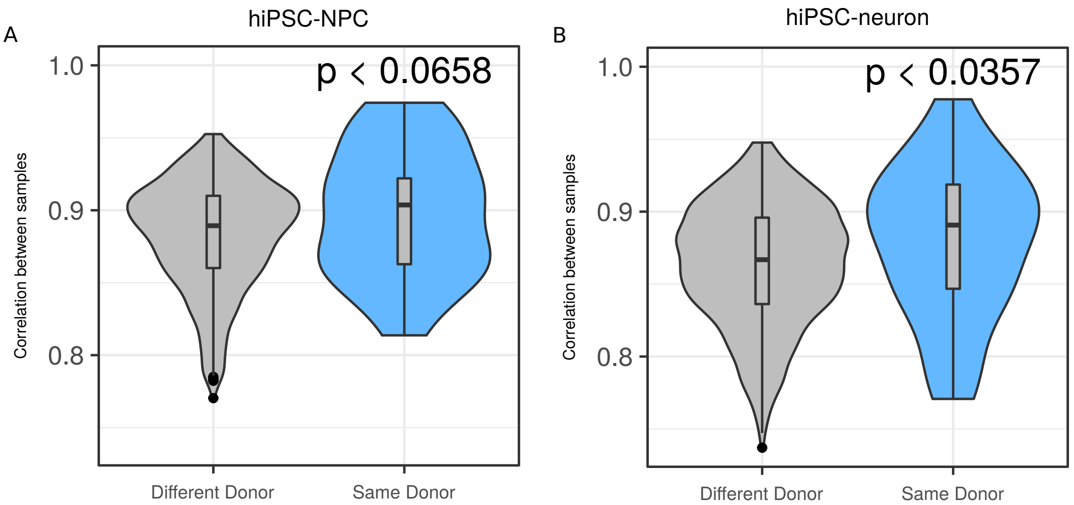

Supplementary Figure 11: **Accounting for fibroblast cell type composition scores increases similarity between RNA-Seq samples from the same donor within each cell type. A,B**) Correlation between samples from different donors compared to the correlation between samples from the sample donor for A) hiPSC-NPCs and B) hiPSC-neurons. P-value indicates one-sided Wilcoxon test.

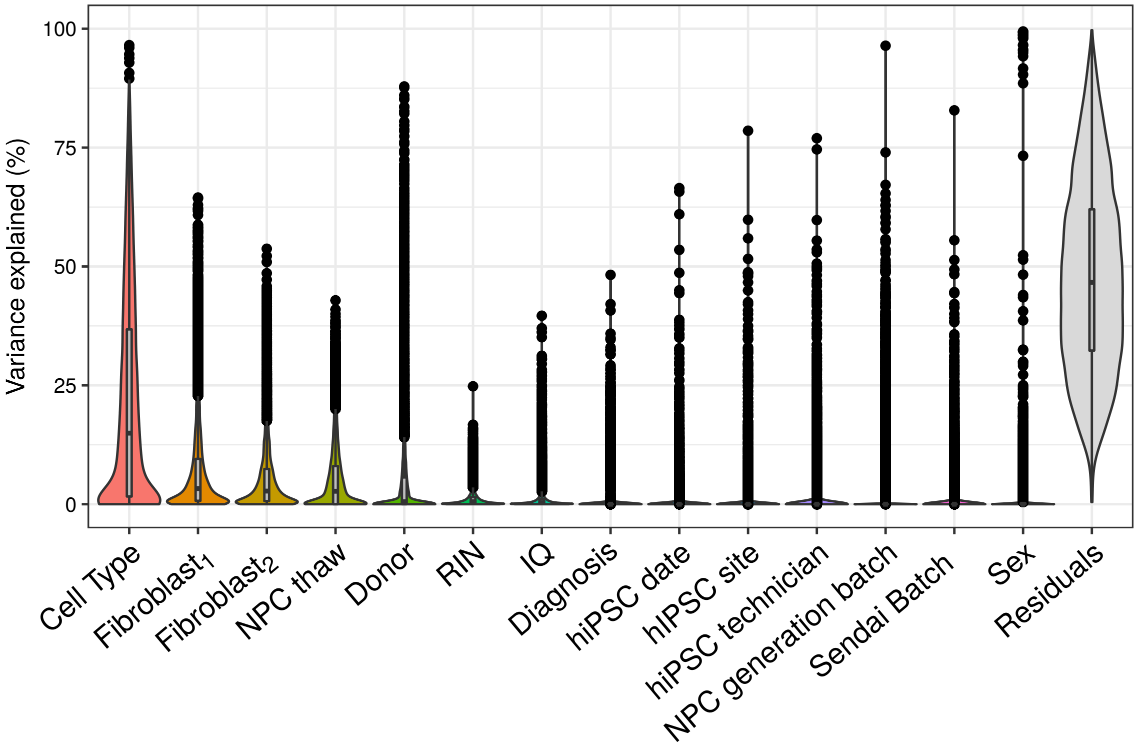

Supplementary Figure 12: Violin plots of the percentage of variance explained by each variable over all the genes for multiple biological and technical sources of variation.

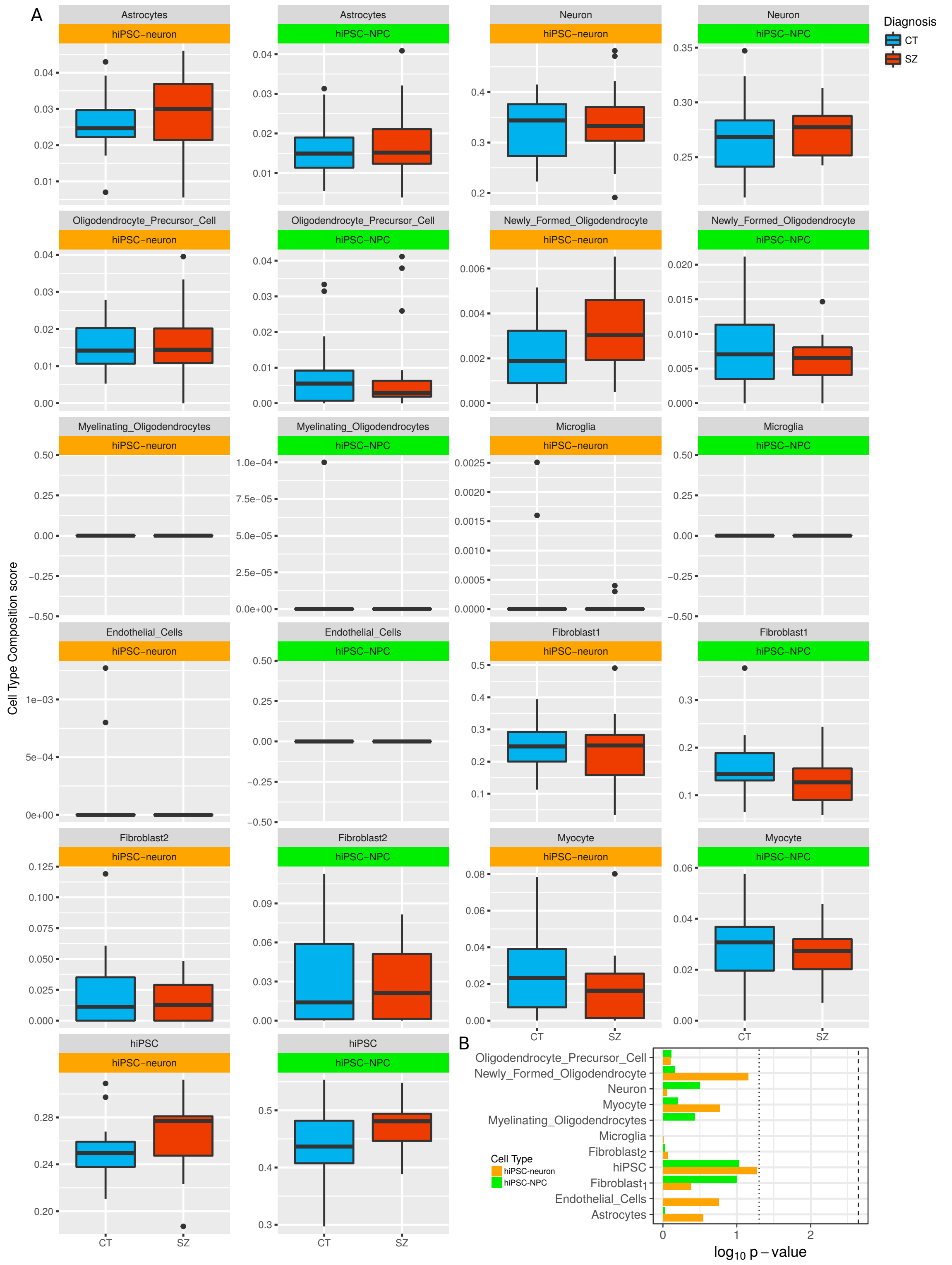

Supplementary Figure 13: No differences in cell type composition scores between cases and controls. **A**) Cell type composition scores stratified by case/control status for hiPSC-neurons and hiPSC-NPCs. **B**) -$\log_{10}$ p-values for hypothesis test (two-sided Wilcoxon) for each boxplot in (A). Dotted line

indicates p-value of 0.05 and dashed line indicates Bonferroni cutoff at 5%. No tests are significant at even the nominal cutoff.

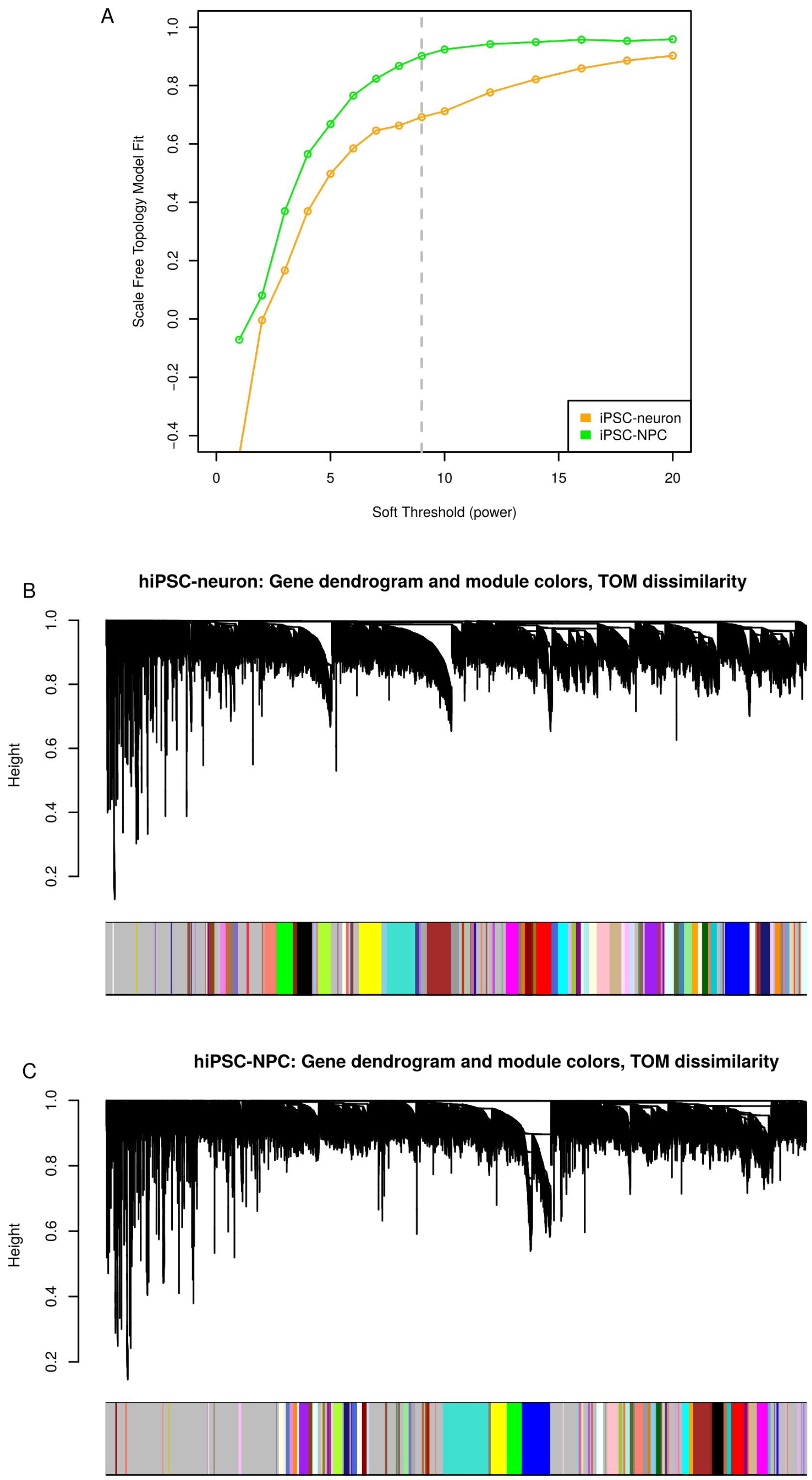

Supplementary Figure 14: Coexpression analysis. A) Metric of scale free network topology for hiPSC-NPC and hiPSC-neuron networks. Dashed line indicates the software threshold of 9 used in the analysis. **B,C**) Dendrogram and module assignments from expression analysis for B) hiPSC-neurons and C) hiPSC-NPCs.

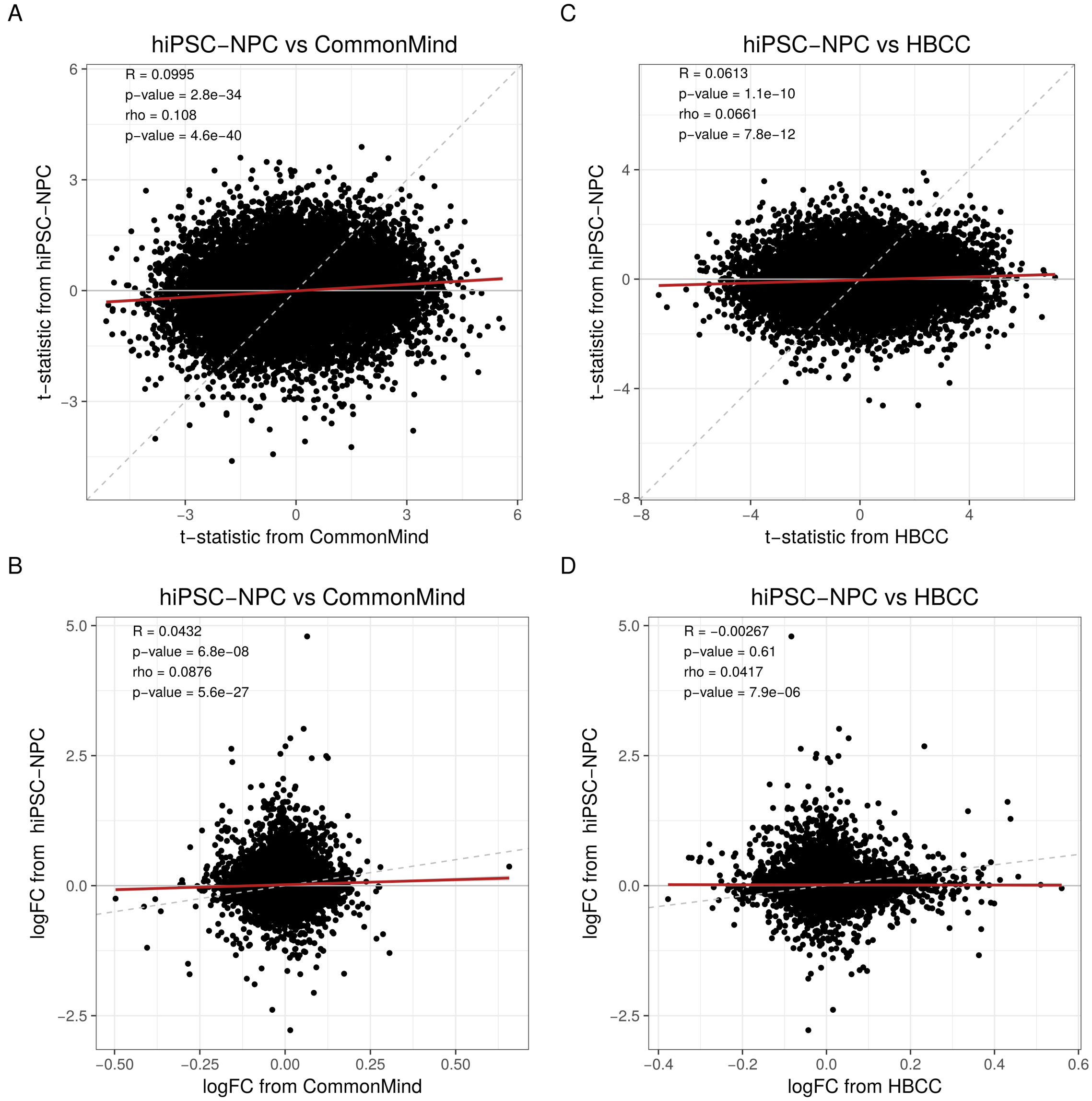

Supplementary Figure 15: Concordance between case/control differential expression results from hiPSC-NPCs from the current study and two adult post mortem cohorts. **A,B**) Concordance between t-statistics from hiPSC-NPCs and A) CommonMind and B) NIMH HBCC cohorts. **C,D**) Concordance between $\log_2$ fold change estimates from hiPSC-NPCs and A) CommonMind and B) HBCC cohorts. Dashed grey line indicates a slope of 1. Dark red line indicates best fit line based on observed data. Correlation between two datasets are summarized in terms of Pearson correlation (R) and Spearman correlation (rho), each with corresponding p-values.

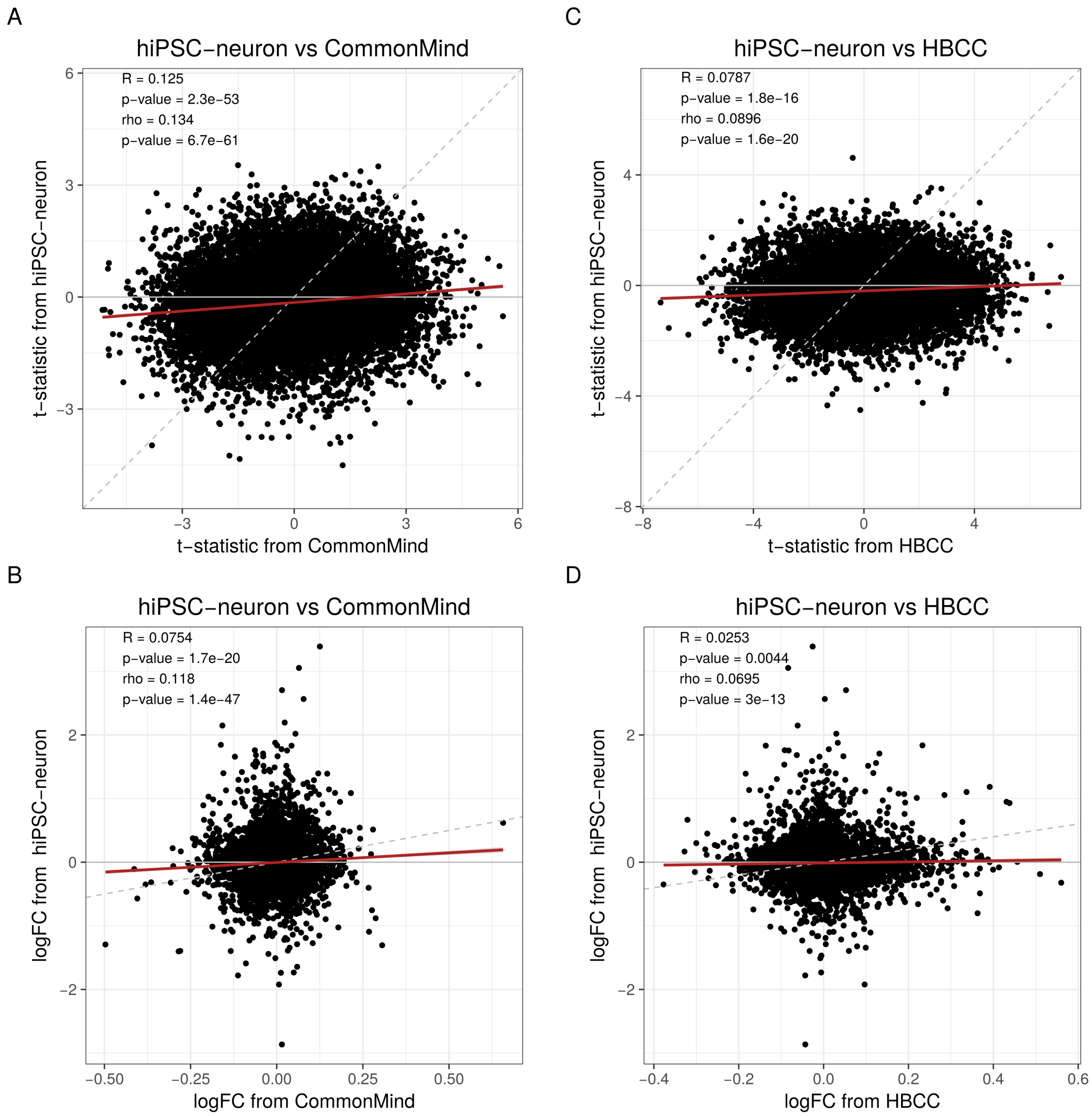

Supplementary Figure 16: Concordance between case/control differential expression results from hiPSC-neurons from the current study and two adult post mortem cohorts. **A,B**) Concordance between t-statistics from hiPSC-neurons and A) CommonMind and B) NIMH HBCC cohorts. **C,D**) Concordance between $\log_2$ fold change estimates from hiPSC-neurons and A) CommonMind and B) HBCC cohorts. Dashed grey line indicates a slope of 1. Dark red line indicates best fit line based on observed data. Correlation between two dataset are summarized in terms of Pearson correlation (R) and Spearman correlation (rho), each with corresponding p-values.

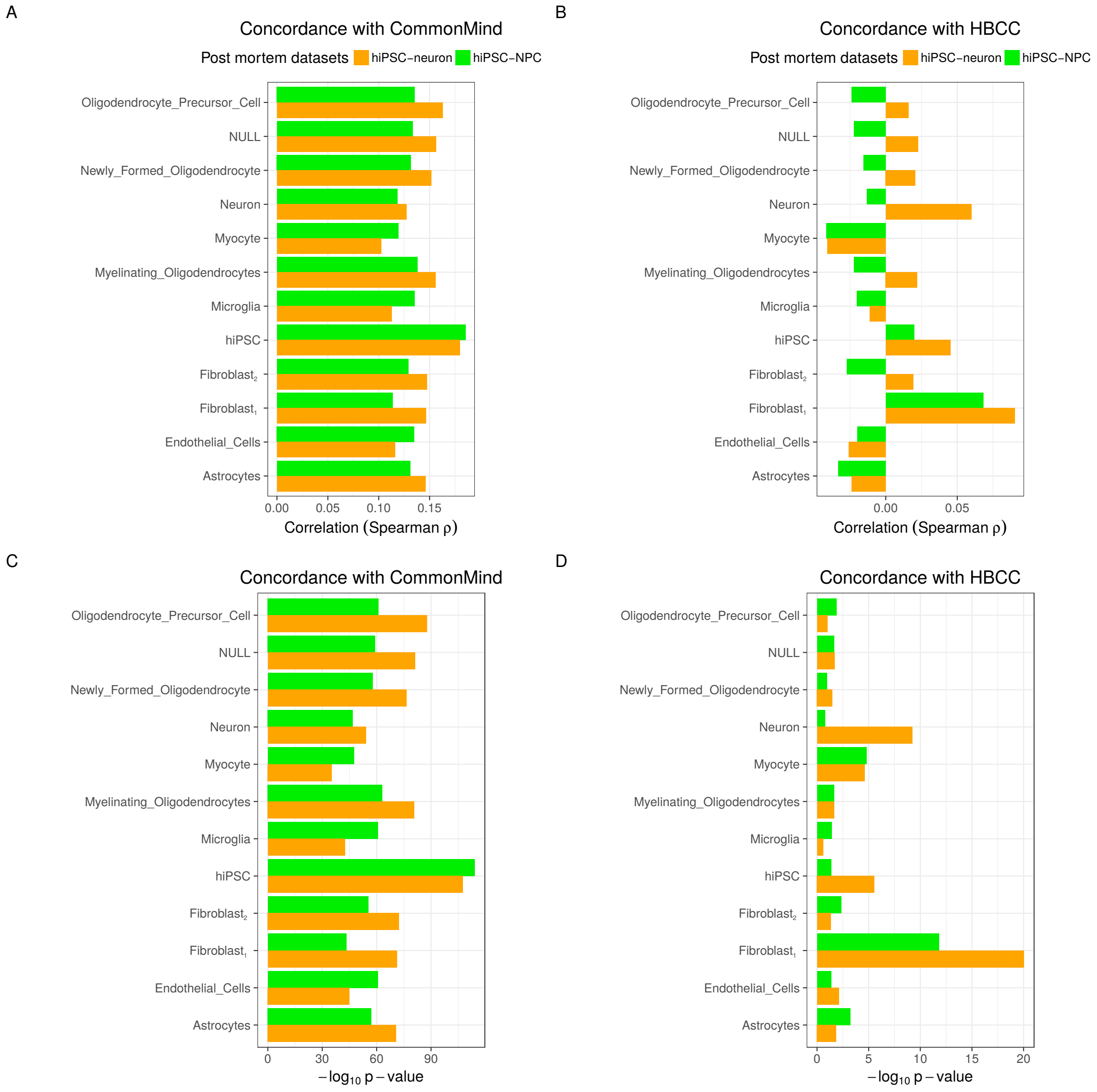

Supplementary Figure 17: Concordance of case/control differential expression signatures between current study and post mortem cohorts depends on correction for cell type composition scores. A,B) Spearman correlation between t-statistics for case/control differential expression analysis from the current study compared to A) CommonMind and B) NIMH HBCC cohorts were cell type composition scores were included as a covariate in the regression model. NULL indicates a model with no score included. Note the large effect of including the fibroblast$_1$ score in the concordance with the HBCC cohort. **C,D**) One-sided hypothesis test for the correlation analysis in the previous panels for C) CommonMind and D) HBCC cohorts.

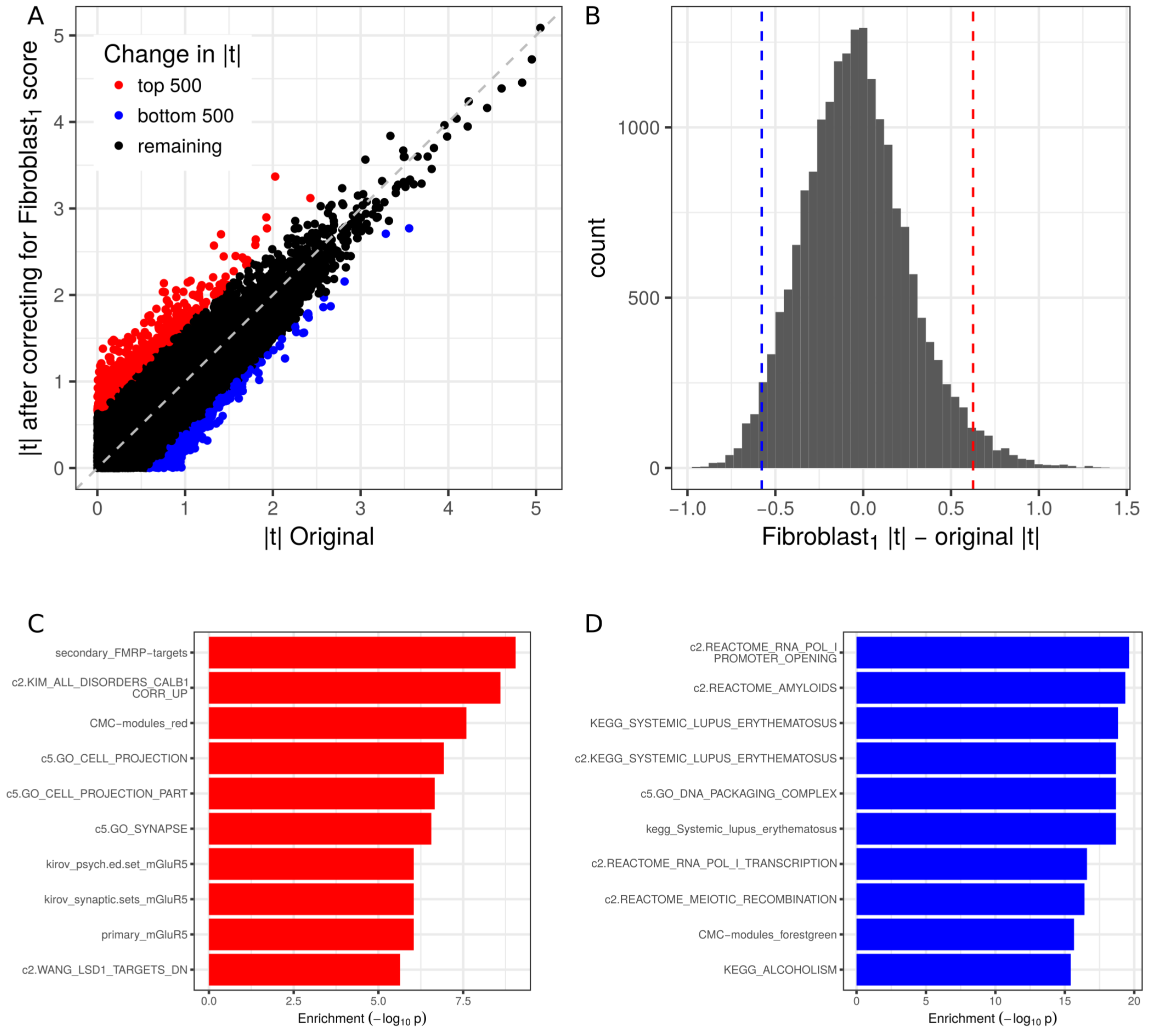

Supplementary Figure 18: Correcting for fibroblast$_1$ cell type composition score in test of case/control differential expression affects specific genes in hiPSC-NPCs. **A**) Comparison of absolute value of t-statistics from differential expression analysis including the fibroblast$_1$ score as a covariate compared to absolute t-statistics omitting it. Dashed line indicates a slope of 1. Genes are colored based on their difference between the two analyses. Red indicates the 500 genes with the greatest increase in the absolute t-statistic and blue indicates the 500 genes with the greatest decrease. The remaining genes are in black. **B**) Histogram of differences in absolute t-statistics from (A). Dashed lines indicate the cutoff for the 500 genes with greatest increase (red) and greatest decrease (blue). **C,D)** Gene set enrichments using a hyper geometric test for the 500 genes with the greatest C) increase and D) decrease of absolute t-statistics.

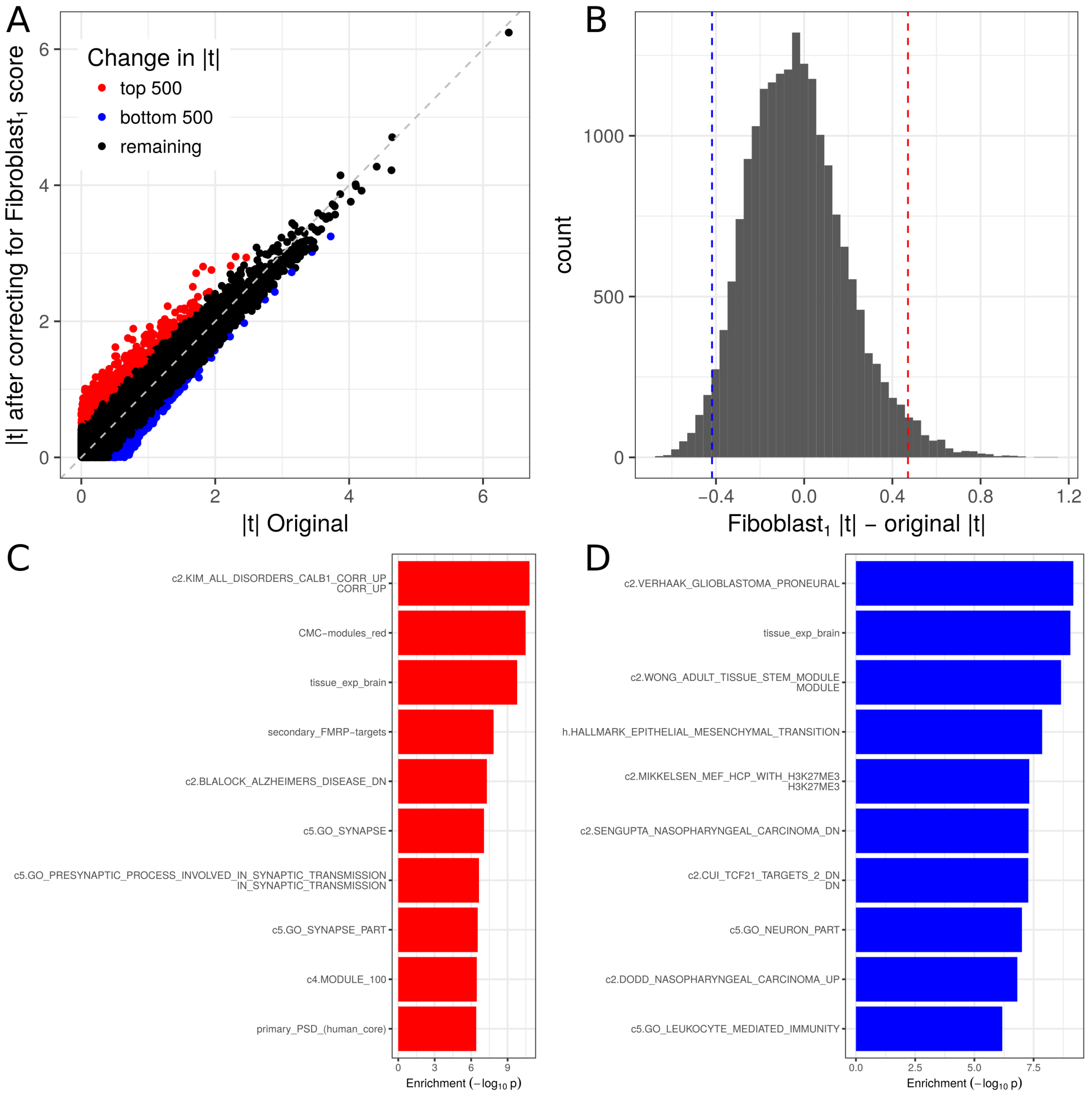

Supplementary Figure 19: Correcting for fibroblast$_1$ cell type composition score in test of case/control differential expression affects specific genes in hiPSC-neurons. **A**) Comparison of absolute value of t-statistics from differential expression analysis including the fibroblast$_1$ score as a covariate compared to absolute t-statistics omitting it. Dashed line indicates a slope of 1. Genes are colored based on their difference between the two analyses. Red indicates the 500 genes with the greatest increase in the absolute t-statistic and blue indicates the 500 genes with the greatest decrease. The remaining genes are in black. **B**) Histogram of differences in absolute t-statistics from (A). Dashed lines indicate the cutoff for the 500 genes with greatest increase (red) and greatest decrease (blue). **C,D)** Gene set enrichments using a hyper geometric test for the 500 genes with the greatest C) increase and D) decrease of absolute t-statistics.

SUPPLEMENTARY NOTE 1

Computing effective sample size

The concept of effective sample size is widely used in statistical genetics to compare the relative power of two or more case/control genome-wide association studies [6]. Assuming that the effect sizes and allele frequencies are the same, the power of each study is determined by the total sample size and the case/control ratio. A balanced study with equal number of cases and control is the most powerful. Because cases tend to be the limiting factor, most studies are unbalanced. In order to compare the power of studies with different sample sizes and case/control ratios, the "effective sample size" (N_e) indicates the sample size of the balanced study with equivalent power. This statistic allows comparisons by putting all studies with different characteristics on the same scale. Moreover, intuition about effective sample size can guide study design to allocate resources to maximize power.

Designing gene expression studies to detect differential expression raises a corollary challenge: how to compare the relative power of studies with different numbers of total experiments and biological replicates, assuming all other factors are equivalent. Biological replicates from the same donor will be correlated because they measure the same underlying biological process. So a comparison of relative power must consider this degree of correlation.

Here we formalize the concept of effective sample size for studies with correlated samples by computing the sample size of a study of independent samples with equivalent power [7]. We start by assuming that all experiments have equal cost (in terms of labor, sequencing, etc.) and relax this assumption below.

Consider a study of k donors with m biological replicates per donor where ρ indicates the correlation between multiple experiments from the sample donor. This corresponds to mk total experiments. Following standard statistical theory of repeated measures study design [8-10] the effective sample size is

$$N_e = \frac{mk}{1 + \rho(m - 1)}$$

Examination of this formula indicates key insights: 1) With 1 biological replicate per donor (m=1), the effective sample size equals the number of donors. 2) The increase in effective sample size obtained by increasing the number of biological replicates (i.e. m) is mediated by the correlation between biological replicates from the same donor (i.e. ρ).

Consider the contribution of each experiment to the power of the study as measured by effective sample size. Letting $N_{total} = mk$ be the total number of experiments, and V be the contribution of each experiment to the effective sample size, then

$$V = \frac{N_e}{N_{total}}$$

$$= \frac{1}{1 + \rho(m-1)}$$

Examination of this formula indicates two key insights: 1) V represents the incremental impact of each successive experiment and is bounded between 0 and 1. 2) The incremental impact is highest when ρ and m are small. The latter point indicates that adding a biological replicate has a larger impact to increase power when there are few replicates or when the correlation between experiments from the same donor is small. When there are already, say, $m = 5$ replicates or ρ is large then the contribution is minimal.

Computing effective sample size when costs are variable

In practice, there are substantial overhead costs for each donor in terms of recruitment, biopsy and hiPSC reprogramming. This overhead makes subsequent experiments from the same donor less expensive than the first experiment. When the total number of experiments in the study is fixed, then

$$k = \frac{N_{total}}{m}$$

is the number of biological replicates per donor. Consider that the cost per experiment varies so that the first experiment from a new donor costs $C1$ units and all subsequent biological replicates cost $C2$ units with $C2 \leq C1$. It follows that the first experiment costs $C1$ units and the sum of all subsequent experiments from the same donor is

$$C2(m-1)$$

and the total cost per donor is

$$C1 + C2(m-1).$$

If the total cost of the study is fixed at C, it follows that the number of donors that can be afforded is

$$k = \frac{C}{C1 + C2(m-1)}$$

A decreased cost of adding biological replicates changes computation of the effective sample size and pushes the calculation to favor increasing biological replicates when the total cost is fixed.

The companion website http://gabrielhoffman.shinyapps.io/design_ips_study/ creates interactive plots showing the effective sample size or the incremental impact of each experiment when either the total cost or number of donors is fixed.

SUPPLEMENTARY REFERENCES

1. Jun, G. *et al.* Detecting and estimating contamination of human DNA samples in sequencing and array-based genotype data. *Am J Hum Genet* **91**, 839-48 (2012).
2. Fromer, M. *et al.* Gene expression elucidates functional impact of polygenic risk for schizophrenia. *Nat Neurosci* **19**, 1442-1453 (2016).
3. Turley, E.A., Veiseh, M., Radisky, D.C. & Bissell, M.J. Mechanisms of disease: epithelial-mesenchymal transition--does cellular plasticity fuel neoplastic progression? *Nat Clin Pract Oncol* **5**, 280-90 (2008).
4. Prescott, S.L. *et al.* Enhancer divergence and cis-regulatory evolution in the human and chimp neural crest. *Cell* **163**, 68-83 (2015).
5. Simoes-Costa, M. & Bronner, M.E. Establishing neural crest identity: a gene regulatory recipe. *Development* **142**, 242-57 (2015).
6. Newton-Cheh, C. *et al.* Common variants at ten loci influence QT interval duration in the QTGEN Study. *Nat Genet* **41**, 399-406 (2009).
7. Blainey, P., Krzywinski, M. & Altman, N. Points of significance: replication. *Nat Methods* **11**, 879-80 (2014).
8. Diggle, P., Heagerty, P., Liang, K.-Y. & Zeger, S. *Analysis of Longitudinal Data*, (Oxford University Press, Oxford, U.K., 2002).
9. Faes, C., Molenberghs, G., Aerts, M., Verbeke, G. & Kenward, M.G. The Effective Sample Size and an Alternative Small-Sample Degrees-of-Freedom Method. *The American Statistician* **63**, 389–399 (2009).
10. Liu, G. & Liang, K.Y. Sample size calculations for studies with correlated observations. *Biometrics* **53**, 937-47 (1997).
