# Supplementary material for: Transcriptional signatures of schizophrenia in hiPSC-derived NPCs and neurons are concordant with post-mortem adult brains
Source: Nat Commun. 2017 Dec 20;8:2225. doi: 10.1038/s41467-017-02330-5 (PMC5738408; doi:10.1038/s41467-017-02330-5)
Supplement: Supplementary file 3 — Description of Additional Supplementary Files [file 41467_2017_2330_MOESM3_ESM.pdf]

File Name: Supplementary Data 1
Description: Clinical and laboratory information about each individual and sample

File Name: Supplementary Data 2
Description: Clinical and laboratory metadata used bioinformatics analysis

File Name: Supplementary Data 3
Description: Quality control statistics for RNA-Seq data

File Name: Supplementary Data 4
Description: Biotype counts for expressed genes

File Name: Supplementary Data 5
Description: Differential expression analysis based on residual Sendai virus expression

File Name: Supplementary Data 6
Description: Gene set enrichments for residual Sendai virus differential expression analysis

File Name: Supplementary Data 7
Description: Coexpression modules and gene set enrichments

File Name: Supplementary Data 8
Description: Differential expression analysis between SZ and controls

File Name: Supplementary Data 9
Description: Gene set enrichments for cell type composition differential expression analysis
